# OnabotulinumtoxinA is a well tolerated and effective treatment for refractory overactive bladder in real-world practice

**DOI:** 10.1007/s00192-020-04423-0

**Published:** 2020-07-27

**Authors:** Rizwan Hamid, Maria-Fernanda Lorenzo-Gomez, Heinrich Schulte-Baukloh, Amin Boroujerdi, Anand Patel, Elisabeth Farrelly

**Affiliations:** 1 University College London Hospitals, Euston Road, London, NW1 2BU UK; 2 University Hospital of Salamanca, Salamanca, Spain; 3 Urologic practice, Department of Urology, Charité University Hospital, Berlin, Germany; 4 Allergan plc, Irvine, CA USA; 5 Allergan plc, Marlow, UK; 6 Södersjukhuset, Stockholm South General Hospital, Stockholm, Sweden

**Keywords:** OnabotulinumtoxinA, Urinary incontinence, Overactive bladder, Quality of life

## Abstract

**Introduction and hypothesis:**

In randomized clinical trials onabotulinumtoxinA was demonstrated to be an effective and well-tolerated treatment for overactive bladder (OAB) with urinary incontinence (UI). However, data reporting onabotulinumtoxinA use in everyday clinical practice are limited. Here, we present the results from a large, first-of-its-kind real-world study in patients with OAB.

**Methods:**

This was a prospective, observational, multinational study (GRACE; ClinicalTrials.gov, NCT02161159) performed in four European countries. Patients (*N* = 504) aged ≥ 18 years with OAB inadequately managed with ≥ 1 anticholinergic received onabotulinumtoxinA per their physician’s normal clinical practice.

**Results:**

Physicians primarily used rigid cystoscopes for onabotulinumtoxinA injection; anesthesia/analgesia was utilized during most treatment procedures. Significant reductions in UI episodes/day from baseline to weeks 1 and 12 were observed as well as in micturition, urgency, and nocturia episodes/day. These improvements in urinary symptoms corresponded to higher scores on the treatment benefit scale at week 12. The use of other OAB medications dropped from baseline to weeks 1 and 12 and was sustained to week 52, which paralleled a reduction in the number of incontinence products used during that time frame. Adverse reactions were reported in 2.6% of patients throughout the study.

**Conclusions:**

In this real-world study, significant improvements in urinary symptoms were seen following onabotulinumtoxinA treatment as early as week 1 and sustained to at least week 12. This was accompanied by a reduced reliance upon incontinence products and reduction in concomitant OAB medication use. OnabotulinumtoxinA was well tolerated with no new safety signals.

**Electronic supplementary material:**

The online version of this article (10.1007/s00192-020-04423-0) contains supplementary material, which is available to authorized users.

## Introduction

Overactive bladder (OAB) is a syndrome characterized by the International Continence Society as “urinary urgency, usually accompanied by frequency and nocturia, with or without urgency urinary incontinence, in the absence of urinary tract infection or other obvious pathology” [1]. An estimated 546 million people worldwide are affected by symptoms consistent with OAB; approximately 423 million have urinary incontinence (UI) [2].

UI as a result of OAB can significantly affect quality of life (QoL) and, as a result of social embarrassment, many patients can suffer from low self-esteem resulting in avoidance and limited social behavior. Many aspects of daily life can be impacted by UI including physical activity, travel, relationships, sexual activity, and quality of sleep [3], and it is also associated with further medical problems such as falls and dermatitis [4, 5]. There is also a substantial direct cost to the patient with UI primarily from the use of absorbent products [6] as well as indirect sequelae costs [7].

First-line pharmacological treatments for OAB with UI include anticholinergic medications and beta-3 agonists. Anticholinergic medications are not always effective over time and can be associated with side effects of dry mouth, constipation, blurred vision [8], and an increased risk of dementia [9]. Beta-3 agonists have been associated with side effects of constipation, dry mouth, headaches, and an increased risk of hypertension [10].

OnabotulinumtoxinA 100 U is approved in many countries for the treatment of OAB with symptoms of urge UI, urgency, and frequency in adults who have an inadequate response to or are intolerant of ≥ 1 anticholinergic [11]. In two large, randomized, placebo-controlled phase 3 studies, onabotulinumtoxinA has been demonstrated to be well tolerated and to improve urinary symptoms and QoL in patients with OAB who were refractory to anticholinergic medications [12, 13].

However, efficacy and safety data with onabotulinumtoxinA for the treatment of OAB in real-world clinical practices are limited, as is information regarding the ongoing concomitant use of other OAB medications and incontinence product use after treatment.

The objective of this study was to evaluate the real-world use of onabotulinumtoxinA up to 1 year in the treatment of patients with idiopathic OAB including the evaluation of changes in urinary symptoms, QoL, incontinence product usage, OAB medication use, and treatment procedures.

## Study design, materials, and methods

This was a large, prospective, observational, non-randomized multinational study (GRACE; ClinicalTrials.gov, NCT02161159) performed in four European countries: Germany (31 sites), Spain (nine sites), Sweden (six sites), and the UK (seven sites).

Patients were enrolled who were aged ≥ 18 years with OAB with UI and who were intolerant to and/or inadequately managed with ≥ 1 anticholinergic medication; all were naive to botulinum toxin type A for the treatment of OAB.

Exclusion criteria included: treatment with any botulinum toxin type A in the prior 18 months for any condition, if the patient was not willing or unable to initiate clean intermittent catheterization (CIC) after onabotulinumtoxinA treatment, or if they were currently taking part in other clinical studies.

Both efficacy and safety analyses were conducted on the safety population (patients who received at least one dose of onabotulinumtoxinA).

Treatment with onabotulinumtoxinA was performed per the physician’s normal clinical practice and based on local summaries of product characteristics (SMPC). Patients were then followed up for 12 months with three scheduled follow-up visits (FU: FU1 approximately 12 weeks, FU2 any time point after FU1, FU3 approximately 12 months after baseline).

Patients could have completed an optional paper diary. Data entered into the diary were analyzed according to predefined documentation time points. Patients could document the following data into the diary at specific time points, which were not necessarily linked with a particular visit: UI, micturition, urgency and nocturia frequency (baseline and weeks 1 and 12 post-injection); Treatment Benefit Scale (TBS, week 12 post-injection only); incontinence product usage (baseline and weeks 12, 20, 28, 36, and 52 post-injection); ongoing OAB medication use (weeks 1, 12, 20, 28, 36, and 52 post-injection).

### Efficacy outcomes

Co-primary end points documented in the patient diary included the reduction in UI episodes/day from baseline (voiding data such as UI episodes were captured for the 3 days before the study time points; week 12 was the primary time point) and the proportion of patients with a positive treatment response at week 12 post-injection on the TBS. A score of 1 or 2 on the TBS, representing “greatly improved” or “improved,” was interpreted as a positive treatment response; a score of 3 indicated “not changed” and 4 indicated “worsened” [14].

Secondary end points included time to reinjection, the proportions of patients with a ≥ 50% and 100% reduction in UI episodes/day from baseline, number of nocturia episodes (voids interrupting night sleep) within 24 h, and absorbent liner/pant/pad use (week 1, over the past week; all other time points, over the past month).

Other end points and additional variables included injection details (time to request reinjection and between request for reinjection and reinjection), number of micturitions and urgency episodes within 24 h, injection procedure including type of analgesia/anesthesia and cystoscope (rigid or flexible) used for onabotulinumtoxinA injection, onabotulinumtoxinA dose, number and location of injection sites, depth of injection, volume injected per site, and number of onabotulinumtoxinA units/ml.

Additionally, data on the use of other OAB medications (weeks 1, 12, 20, 28, 36, and 52) were captured.

### Retreatment

Retreatment with onabotulinumtoxinA was based on patient request and physician judgment at the follow-up visits when this was in line with the local SMPC and the treating physician’s normal clinical practice.

### Safety

Adverse events (AEs; defined as any untoward medical occurrence in a patient using onabotulinumtoxinA which does not necessarily have to have a causal relationship with this treatment) and adverse reactions (ARs; defined as an event for which a causal relationship to the procedure was rated as reasonably possible) were recorded up to 12 months post-treatment.

### Statistics

Summary statistics were provided for continuous variables; frequencies and percentages in each category were provided for categorical data; 95% confidence intervals (CIs) were calculated for the primary efficacy end points “proportion of subjects with positive treatment response at week 12” and “reduction of UI episodes between baseline and week 12.”

The one-sided Wilcoxon signed rank test was used to indicate a reduction in the number of UI episodes from baseline to week 12 (actual and percent reduction). The Wilcoxon signed rank test was also used to evaluate the changes from baseline to each documented time point in the mean number of micturitions, mean number of urgency episodes, mean number of nocturia episodes, and number of incontinence products used (sum of all products).

The McNemar test was used to evaluate the changes between week 1 and week 12 in the proportions of patients with ≥ 50% and 100% reduction from baseline in UI episodes/day.

Statistical testing was exploratory and performed on the two-sided 0.05 level of significance.

Data entered into the diary were analyzed according to predefined time points. Diary data on daily UI, micturitions, urgency, and nocturia episodes were averaged for each patient over the last 3 consecutive days before baseline, week 1, and week 12.

Missing data were not imputed. If the onset date of an AE was missing or partially missing, the AE was considered an AR.

This study was conducted in accordance with the ethical principles that have their origin in the Declaration of Helsinki and that are consistent with good clinical practice and the applicable regulatory requirement(s). The study protocol was approved at each study site by an ethics committee or institutional review board, and all patients provided signed informed consent.

## Results

### Patients

Of the 515 patients who signed the informed consent form, 11 were not treated. The remaining 504 patients who were treated with onabotulinumtoxinA comprised the safety analysis (Supplementary Table 1). The majority of these patients (59.3%, *n* = 299) completed the study; 205 patients (40.7%) discontinued the study prematurely. Most of these patients (29.4%, *n* = 148) were “lost to follow-up.”

Baseline demographics and disease characteristics are shown in Table 1. The study population was primarily women, on average in their mid-60s, who were living independently at home. The mean time since initial onset of symptoms was approximately 7 years.

**Table 1:** Baseline demographics

Parameter	(*N* = 504)
Age, years, mean ± SD	63.3 ± 14.1
Age group, *n* (%)	
≥ 18 and < 30 years	15 (3.0)
≥ 30 and < 40 years	20 (4.0)
≥ 40 and < 50 years	43 (8.5)
≥ 50 years	418 (82.9)
Gender, *n* (%)	
Female	428 (84.9)
Circumstances of life, *n* (%)	
At home - independent	476 (94.4)
At home with nursing care	26 (5.2)
Nursing home	2 (0.4)
Time since initial onset of symptoms, months, mean ± SD	84.6 ± 80.6
At least one OAB medication documented at baseline, *n* (%)^a^	469 (93.1)
Patients intending to continue using OAB medications, *n* (%)	52 (10.3)
OAB medication use at baseline, *n* (%) Duration of medication use before onabotulinumtoxinA treatment median [min, max], weeks	
Anticholinergics	430 (85.3) 24 [0, 552]
Solifenacin	200 (39.7) 36 [0, 356]
Trospium	167 (33.1) 24 [1, 384]
Fesoterodine	125 (24.8) 24 [1, 384]
Oxybutynin (skin patches)	65 (12.9) 12 [0, 432]
Darifenacin	53 (10.5) 44 [2, 241]
Tolterodine	44 (8.7) 24 [0, 552]
Oxybutynin (oral)	26 (5.2) 53 [0, 328]
Others	33 (6.5) 24 [1, 480]
Beta-3 adrenergic agonists	183 (36.3) 17 [0, 204]
Mirabegron	183 (36.3) 16 [0, 204]
Others	2 (0.4) 92 [24, 160]
Tricyclic antidepressants	13 (2.6) 96 [4, 480]
Doxepin	1 (0.2) 44 [44, 44]
Imipramine	0 0
Others	12 (2.4) 96 [4, 480]
Main symptom, *n* (%)	
Urge incontinence	312 (61.9)
Urgency (desire to void)	109 (21.6)
Frequent urination	44 (8.7)
Nocturnal urination	18 (3.6)
Pain	7 (1.4)
Other	14 (2.8)
Unknown	0

^a^Patients could be on more than one OAB medication

*OAB* overactive bladder, *SD* standard deviation

At baseline the use of ≥ 1 OAB medication was documented in 469/504 patients (93.1%). Most patients (430/504, 85.3%) used anticholinergics (Table 1). Of those patients on anticholinergics the median duration of treatment before enrollment into the study was 24 weeks. The use of the beta-3 adrenergic agonist mirabegron was documented in 183 patients (36.3%) at baseline, and the median duration of mirabegron treatment prior to entering the study was 16 weeks.

Symptoms described by the patients as most bothersome were urge incontinence followed by urgency (the desire to void), frequent urination, nocturnal urination, and pain (Table 1).

### OnabotulinumtoxinA injection procedure

Details of the injection procedure are shown in Table 2. Overall, patients received a mean (standard deviation [SD]) of 1.3 (0.5) injections throughout the study (median [min, max]: 1.0 [1, 4] injections), and in total, there were 639 treatment sessions in the 504 patients. Anesthesia or analgesia was used in all but 2 (0.3%) treatment sessions, most frequently intravesical instillation, followed by local anesthetic gel, general anesthesia, and sedation. At most treatment sessions (542/639 [84.8%]), only one type of anesthesia/analgesia was used; a combination of methods was used during 95 of the treatment sessions (14.9%).

**Table 2:** Injection methodology

	Treatment sessions, *n* (%)^a^ (*N* = 639)
Type of anesthesia/analgesia^b^	
Intravesical instillation	239 (37.4)
Local anesthetic gel	205 (32.1)
General anesthesia	173 (27.1)
Sedation	117 (18.3)
None	2 (0.3)
Type of cystoscope	
Rigid	543 (85.0)
Flexible	96 (15.0)
Injection sites used	
Limited to trigone vesicae	5 (0.8)
Exclusions of trigone vesicae	222 (34.7)
Distributed	412 (64.5)
Depth of injection^b^	
Submucosal	538 (84.2)
M. detrusor vesicae	144 (22.5)

^a^Percentages are based on the total number of treatment sessions performed in all patients in the safety analysis set

^b^Multiple answers were possible

Physicians used rigid cystoscopes for most treatment procedures (85.0%). Across all treatment sessions (*N* = 639), the mean (SD) number of injection sites was 17.3 (5.2). The number of injection sites (mean [SD]) was highest at the first treatment session (*n* = 504, 17.5 [5.1]), decreasing with each subsequent retreatment (retreatment 1, 17.0 [5.3], *n* = 122; retreatment 2, 13.5 [5.2], *n* = 12; retreatment 3, 10.0, *n* = 1).

In the majority of treatment sessions the injection sites were distributed between the trigone and trigone-sparing regions (64.5%). In a very small number of treatment sessions injections were limited to the trigone (0.8%) and in the remainder the trigone was excluded (34.7%). OnabotulinumtoxinA was more frequently injected into the submucosal layer (84.2%) than the M. detrusor (22.5%).

The median total dose of onabotulinumtoxinA was 100 U for each treatment session. A mean ± SD total volume of 12.3 ± 6.2 ml (median: 10 ml; *n* = 504) and 8.7 ± 4.8 units/ml onabotulinumtoxinA were used per patient session.

### Co-primary efficacy outcomes

**Fig. 1:**
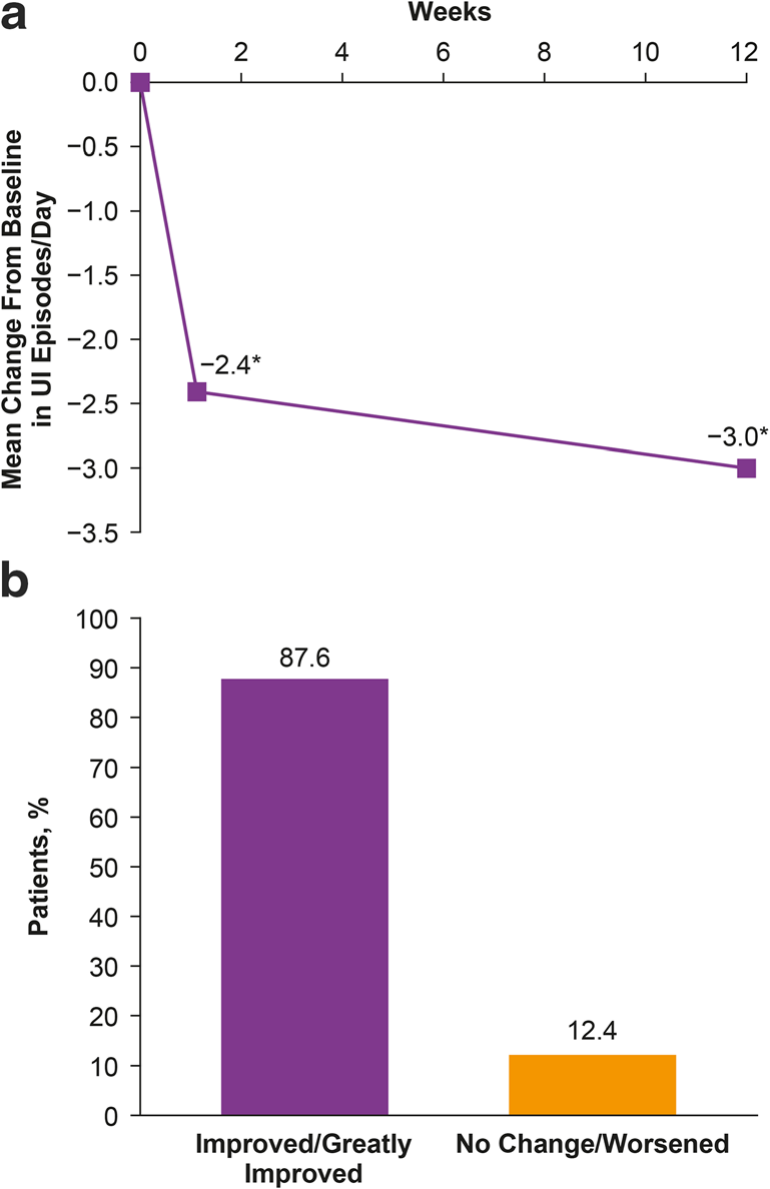
**a** Change from baseline in UI episodes over time. **b** Proportion of patients with a positive (improved/greatly improved) response and no change/worsened response on the TBS at week 12. *Statistically significant (*p* < 0.001 vs. baseline). TBS, treatment benefit scale; UI, urinary incontinence

Reductions in UI episodes/day were observed as early as week 1 (mean ± SD change from baseline, −2.4 ± 3.4, *p* < 0.001 versus baseline, Fig. 1a). This improvement from baseline was sustained until the primary time point of week 12 (−3.0 ± 3.9, *p* < 0.001 versus baseline). This translated into reductions in UI episodes/day of −46.9 ± 64.8% at week 1 and − 61.3 ± 58.6% at week 12 (*p* < 0.001 versus baseline for both time points).

TBS data were available for 347 patients. A positive treatment response on the TBS at week 12 was seen in 87.6% of these patients (Fig. 1b).

### Secondary and other efficacy outcomes

#### Urinary symptoms

Overall, 25.5% of patients became completely continent (i.e., 100% reduction in UI episodes/day) within 1 week after treatment with onabotulinumtoxinA; this increased to 41.8% at week 12 (*p* < 0.001 versus week 1). In addition, a ≥ 50% reduction in UI episodes/day was seen in 60.7% of patients at week 1, increasing to 73.9% at week 12 (*p* = 0.0016 versus week 1). Significant reductions from baseline (*p* < 0.001) were also seen in daily episodes of urgency (baseline: 7.6, week 1: 4.2, week 12: 2.5), nocturia (baseline: 2.6, week 1: 1.8, week 12: 1.2), and micturition (baseline: 11.2, week 1: 8.9, week 12: 7.7) (Supplementary Fig. 1).

#### Incontinence product usage

**Fig. 2:**
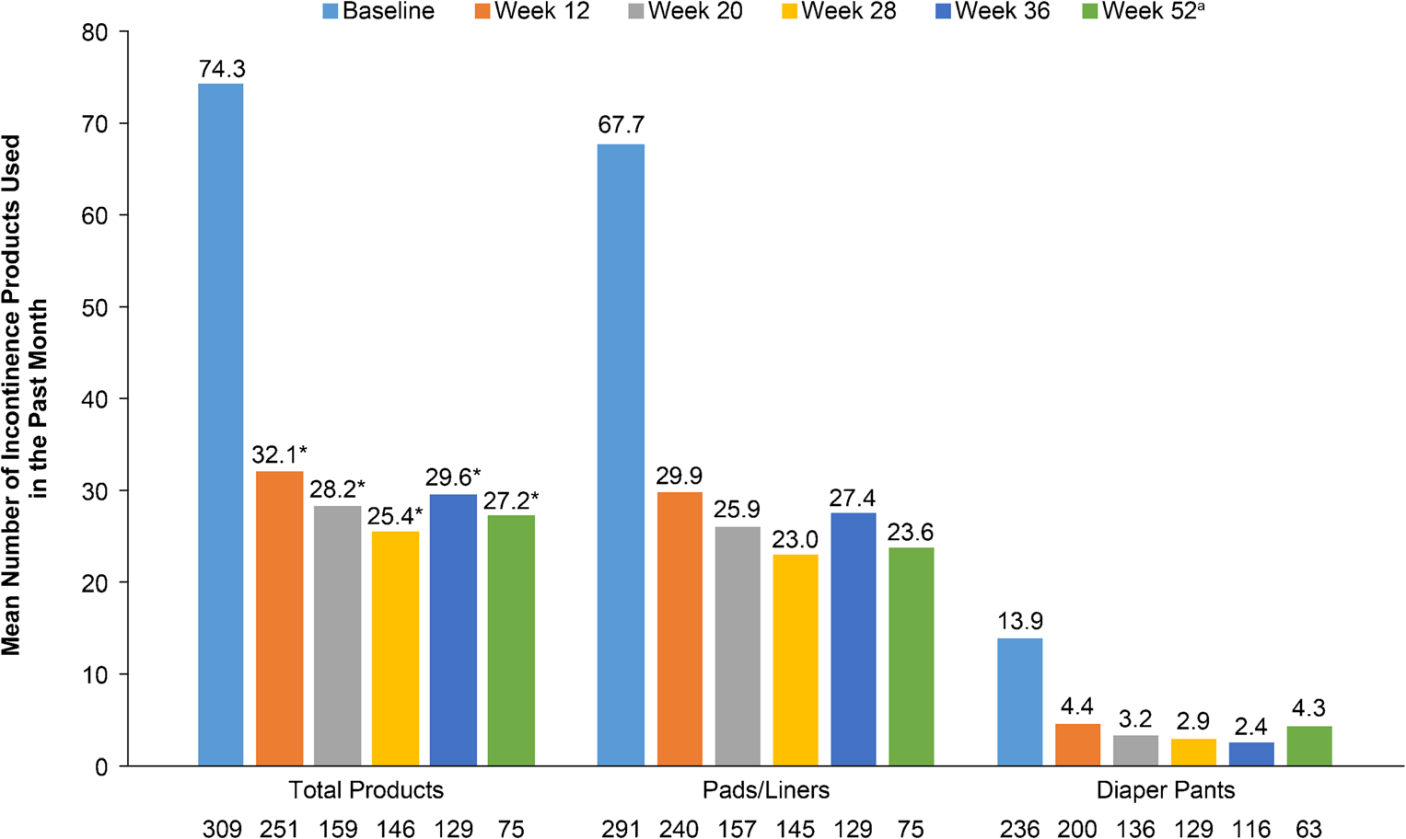
Reduction in incontinence product use from baseline over time. ^a^Week 52: only patients without reinjection of onabotulinumtoxinA. ^*^Statistically significant (*p* < 0.05) vs. baseline

The mean number of incontinence products (total of pads/liners and diaper pants) used over the prior month decreased from baseline (74.3) to week 12 (32.1, *p* = 0.001) and remained constant over the remainder of the study out to week 52 (27.2, *p* = 0.001, Fig. 2). More specifically, pad/liner usage more than halved from a mean of 67.7 in the month prior to baseline to 29.9 and 23.6 in the month prior to week 12 and week 52, respectively. Over the same time period diaper pant usage decreased from a mean of 13.9 during the month prior to baseline to 4.4 and 4.3 during the month prior to weeks 12 and 52, respectively.

#### OAB medication use

**Fig. 3:**
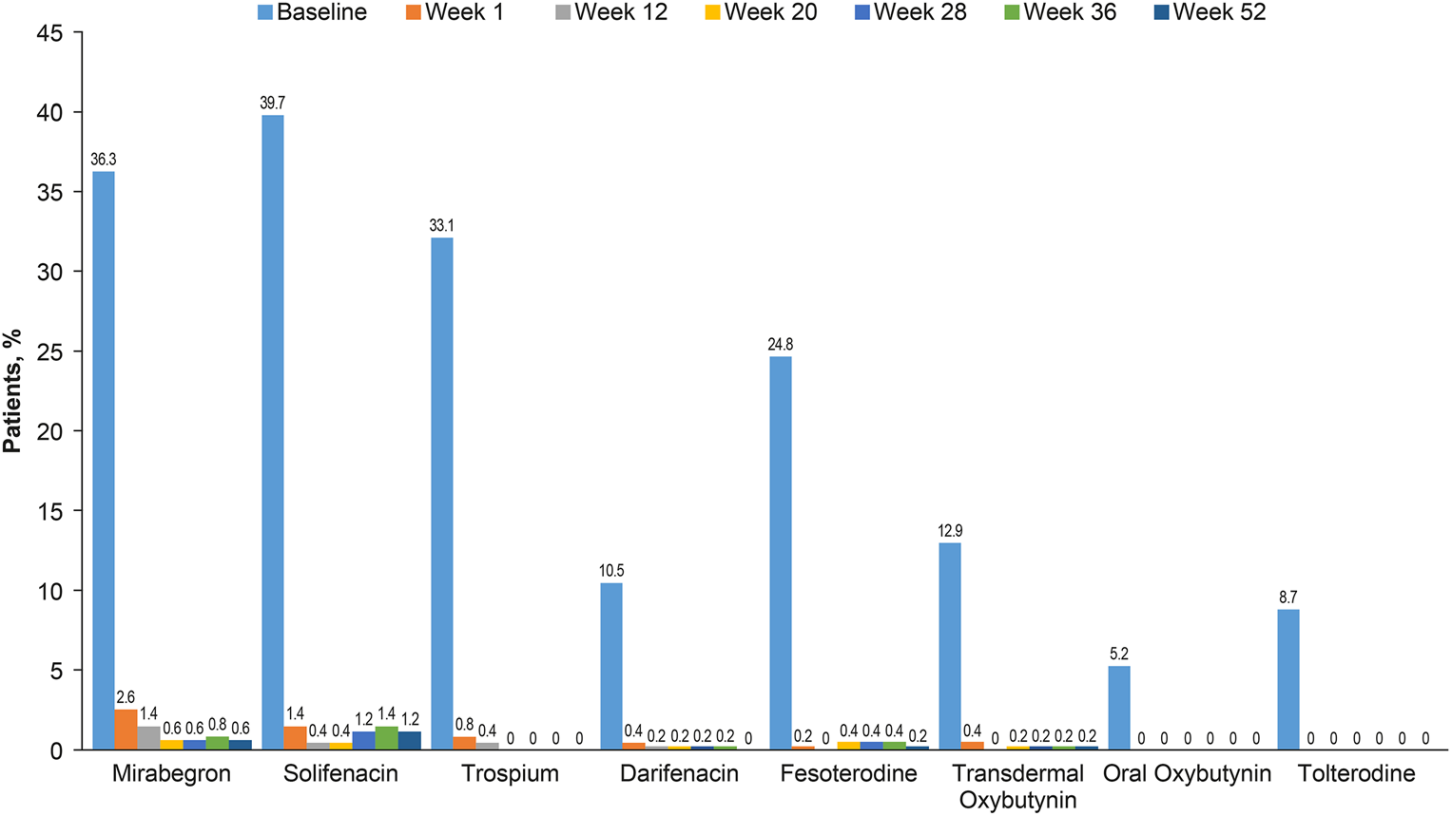
OAB medication use: Baseline: at enrollment; week 1: during the last week; week 12, 20, 28, 36, 52: during the last 4 weeks. Data at week 52 were only in patients without reinjection of onabotulinumtoxinA. Patients could receive more than one medication

At baseline, 52 of 504 patients (10.3%) documented that they intended to continue to use other OAB medications following onabotulinumtoxinA treatment (Table 1). However, during week 1 the number of patients actually using other OAB medications had decreased to approximately half of that number (29 patients, 5.8%), by week 12 only 12 patients (2.4%) had used OAB medications in the past month, and at week 20 this dropped to 9 patients (1.8%). By week 28 the number of patients who used OAB medications within the last month increased slightly to 13 (2.6%), and this was sustained to week 52. Specifically, this was a result of four additional patients using solifenacin. Other anticholinergics were less frequently used. The use of each individual OAB medication up to week 52 is shown in Fig. 3. Treatment with the beta-3 adrenergic agonist mirabegron was ongoing in 13 patients (2.6%) at week 1, 7 (1.4%) at week 12, and had dropped to 3 patients (0.6%) at week 28 after treatment.

#### Retreatment

Overall, 122 patients (24.2%) received retreatment in this 12-month study, 110 had only one retreatment during the study, 11 had two retreatments, and one had three retreatments. The median time to the first retreatment was 298 days (approximately 10 months), and the median time to patient request for first retreatment was 218 days (approximately 7 months). The median time between the patient request for first retreatment and actual first retreatment was 33 days.

#### Safety

Throughout the first treatment cycle, a total of 57 AEs were reported in 38/504 patients (7.5%); 9 patients experienced a total of 17 AEs that were classified as serious (Table 3). During the study one fatality occurred (cerebrovascular accident); the patient death was not considered related to the treatment. Overall, 17 ARs (all non-serious) were reported by 13/504 (2.6%) patients: 7 reported mild ARs, 5 moderate, and 1 severe. Urinary retention was the most frequent AR and was reported in five patients (1.0%), one of which was severe based on the physician’s assessment; this patient also experienced two mild ARs (dysuria and micturition urgency). For each of these five patients, post-treatment CIC was utilized. Overall, 441/639 (69.0%) treatments were performed with antibiotic prophylaxis. Urinary tract infection was reported in two patients (0.4%); both patients had received prophylactic treatment with antibiotics.

**Table 3:** Adverse events and adverse reactions

Patients	Safety population (*N* = 504)	
	Adverse events	Adverse reactions
Total number of events, *n*	57	17
Number of serious events	17	0
Number of non-serious events	40	17
Patients with events, *n* (%)	38 (7.5)	13 (2.6)
Patients with non-serious events	33 (6.5)	13 (2.6)
Patients with serious events	9 (1.8)	0
Deaths^a^	1 (0.2)	0
Patients with non-serious events(s) by intensity, *n* (%)		
Mild	13 (2.6)	7 (1.4)
Moderate	16 (3.2)	5 (1.0)
Severe	2 (0.4)	1 (0.2)
Not available	2 (0.4)	0
Urinary-related events with a frequency ≥ 0.4%, *n* (%)		
Urinary retention^b^		5 (1.0)
Urinary tract infection^c^		2 (0.4)

Adverse reactions were defined as events for which a causal relationship to onabotulinumtoxinA treatment was rated as reasonably possible

^a^Cerebrovascular accident

^b^CIC was initiated in all five patients

^c^The two patients with urinary tract infection had received antibiotic prophylaxis orally on the day of injection

*CIC* clean intermittent catheterization

## Discussion

This real-world study is the largest prospective, multinational, observational study performed to date and was designed to evaluate the efficacy and safety of treatment with onabotulinumtoxinA in patients with OAB as well as the treatment methodologies used by the physicians in their day-to-day clinical practices.

When treating patients with OAB with onabotulinumtoxinA in their clinical practices, the majority of physicians in this study administered a dose of 100 U while using a rigid cystoscope for injection. Local anesthesia instillation into the bladder was the most commonly employed anesthesia.

Physicians appear to have refined their process versus the label with injections in < 20 sites, mainly submucosally and distributed between both the trigone and trigone-sparing regions. Moreover, a small study of patients with idiopathic detrusor overactivity (*n* = 22) or neurogenic detrusor overactivity (*n* = 23) demonstrated efficacy and tolerability with as little as one to three intradetrusor injections. These results were comparable to those seen using the established technique of 20 intradetrusor injections [15]. As physicians gain more experience with the use of onabotulinumtoxinA they can learn how to optimize the administration and effectiveness to better treat OAB patients with their bothersome symptoms.

In this study, all patients had previously failed treatment with anticholinergic therapy, and by the time they received onabotulinumtoxinA many had been receiving anticholinergics for 24 weeks, with approximately 15% discontinuing those medications prior to baseline.

After onabotulinumtoxinA treatment, significant improvements were observed in UI as early as within week 1 (−2.4 episodes/day). These improvements were sustained to at least week 12 (−3.0) and were similar to those observed in phase 3 trials at week 12 (−2.7 to −3.2) [12, 13, 16]. While all patients were required to have UI to be included in this study, more than one in five reported that urgency was their primary symptom, indicating that UI was less bothersome to them than the urge to void. In this study, not only was UI frequency improved but also other urinary symptoms including urgency, suggesting improvement in other pertinent symptoms in this cohort of patients. The improvement in urinary symptoms was accompanied by an improvement in QoL measured by TBS; most patients expressed a positive response 12 weeks following treatment. This was higher than the response observed in previous clinical studies (62.8% and 60.8%) [12, 13]. There was also a reduced reliance upon incontinence products which would be associated with significant cost savings. The Prospective Urinary Incontinence Research study, which looked at the economic burden in women with UI in various European countries (Germany, Spain, UK/Ireland), showed the cost of incontinence products was a large contributor to the overall cost of UI management with more than half of the patients paying out of pocket [17].

The reduction in ongoing medication use observed during this study could indicate that onabotulinumtoxinA-treated patients may decrease or even discontinue their prior OAB medications as they are achieving sufficient control with onabotulinumtoxinA alone. This also emphasizes the benefits of treating patients sooner with onabotulinumtoxinA as opposed to cycling on oral medications. The slight increase in solifenacin use observed between weeks 24 and 28 is consistent with the expected duration of onabotulinumtoxinA and the time at which patients requested retreatment in this study. The reduction in medication use aligns with the reduction in incontinence pad usage, further confirming the efficacy of onabotulinumtoxinA and potentially a better cost-effective option than oral therapy for OAB patients as has been suggested in previous studies [18–20].

It is noteworthy that there was a delay of about 1 month between a patient’s request for retreatment and the patient actually receiving retreatment. This reinforces how important it is to treat patients optimally so they do not experience full reoccurrence of their symptoms. Suffering through multiple “peaks” and “troughs” can be highly discouraging for the patients. To avoid this, healthcare providers may wish to take a proactive approach with patients and schedule follow-up appointments/retreatments every 6 to 7 months.

No new safety signals related to onabotulinumtoxinA treatment were identified, and the rates of AEs were low. In particular, the incidence of urinary retention and urinary tract infections, the definitions of which were discussed and approved by the US Food and Drug Administration, throughout the 52 weeks was very low (1.0% and 0.4%, respectively). In the phase 3 RCTs over the 24 weeks of these studies urinary retention was 5.8% to 6.9% and urinary tract infection was 24.1% to 25.5% [12, 13, 16]. While direct comparisons cannot be made the differences between this real-world study and the data from the phase 3 studies may in part reflect the more conservative criteria that were developed for the purpose of those clinical trials. Specifically, in the RCTs urinary retention was defined as a post-void residual urine volume ≥ 200 ml that required CIC, and urinary tract infection was defined as both a positive urine culture (bacteriuria count of > 10^5^ colony forming units/ml) and leukocyturia (> 5 cells per high power field) regardless of clinical symptoms. In this real-world study, no such specific criteria were required for documentation of urinary retention or urinary tract infection which was diagnosed under the discretion of the physician, and may be reflective of real-world AEs associated with onabotulinumtoxinA treatment. Results from a retrospective review of electronic health records from 99 women evaluated 2 weeks after receiving onabotulinumtoxinA 100 U demonstrated CIC was required following 1.6% of injections (3/187). As with this current study, it was suggested that the low CIC rate was reflective of the less rigorous criteria for CIC initiation compared with prior studies [21]. Previous studies have demonstrated that antibiotic prophylaxis can mitigate the risk for developing urinary tract infections following onabotulinumtoxinA treatment [22, 23], and for the majority of injections performed in this real-world study patients did receive antibiotic pretreatment.

Limitations of this study may be associated with the observational nature of the study design, such as the lack of randomization and a comparator group. In addition, while some patients appear to be restarting other OAB medications after about 7 months following initial treatment, no data are available to specifically identify which patients have or have not been retreated with onabotulinumtoxinA so as to better determine if the retreated group of patients was not reinitiating OAB medications. In contrast to clinical trials, there were no exact evaluation time points in this non-interventional study, as data were collected by the physicians from all clinic visits that occurred as per normal practice in the 12 months that followed the baseline visit.

## Conclusion

The results from this real-world trial may better inform physicians about what to expect following onabotulinumtoxinA treatment for OAB in their day-to-day clinical practice. The data add to the growing body of evidence that onabotulinumtoxinA can improve urinary symptoms and QoL in most patients with OAB symptoms who had not seen lasting benefit with other OAB medications [24]. In addition, the reduction in oral medications and incontinence product usage may provide significant cost savings for the patients.

The low rates of urinary retention and urinary tract infection seen in this study suggest that onabotulinumtoxinA treatment may be better tolerated in clinical practice than previously suggested from the results reported for RCTs.

Physicians may wish to consider proactively scheduling a structured time frame for follow-up/retreatment to avoid a lag time between the patients requesting and actually receiving retreatment, thus preventing a potential recurrence of OAB symptoms.

Given how well tolerated onabotulinumtoxinA was over the 52 weeks of this study, and the sustained efficacy with regard to UI and QoL out to at least 12 weeks, healthcare providers may wish to consider moving patients to onabotulinumtoxinA sooner as opposed to cycling on oral medications.

## Electronic supplementary material

ESM 1 (PDF 105 kb)

Supplementary Fig. 1 Reduction in urgency, nocturia, and micturition episodes/day after onabotulinumtoxinA treatment. *Statistically significant (*p* < 0.001) vs. baseline (PNG 1033 kb)

High-resolution image (TIF 933 kb)
