# Supplementary material for: OnabotulinumtoxinA is a well tolerated and effective treatment for refractory overactive bladder in real-world practice
Source: Int Urogynecol J. 2020 Jul 27;32(1):65–74. doi: 10.1007/s00192-020-04423-0 (PMC7788019; doi:10.1007/s00192-020-04423-0)
Supplement: Supplementary file 1 — (PDF 105 kb) [file 192_2020_4423_MOESM1_ESM.pdf]

Supplementary materials

Supplementary Table 1 Patient disposition

Patients, n (%)	Safety analysis population (*N* = 504)
Baseline documentation available	504 (100)
Completion of the study	299 (59.3)
Early discontinuation	205 (40.7)
Reasons for early discontinuation	
Lost to follow-up	148 (29.4)
Patient’s decision	2 (0.4)
Patient safety consideration	1 (0.2)
Other	7 (1.4)
Missing reasons	47 (9.3)
