# Supplementary figures and images for: OnabotulinumtoxinA is a well tolerated and effective treatment for refractory overactive bladder in real-world practice
Source: Int Urogynecol J. 2020 Jul 27;32(1):65–74. doi: 10.1007/s00192-020-04423-0 (PMC7788019; doi:10.1007/s00192-020-04423-0)

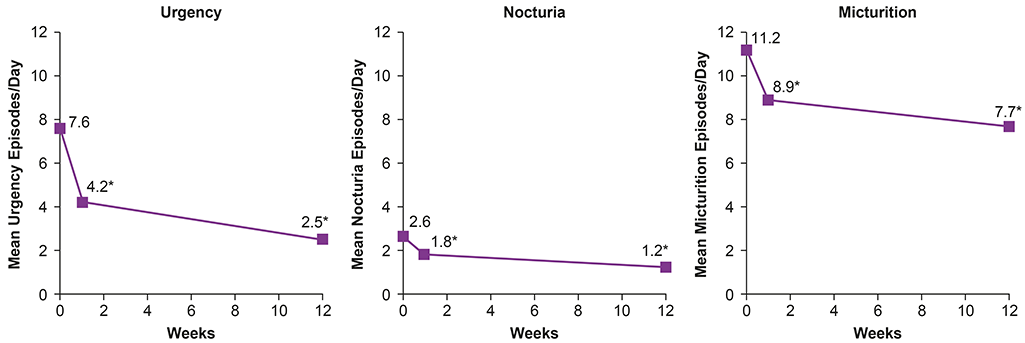

Supplement: Supplementary file 2 — Reduction in urgency, nocturia, and micturition episodes/day after onabotulinumtoxinA treatment. *Statistically significant (p < 0.001) vs. baseline (PNG 1033 kb) [file 192_2020_4423_Fig4_ESM.png]

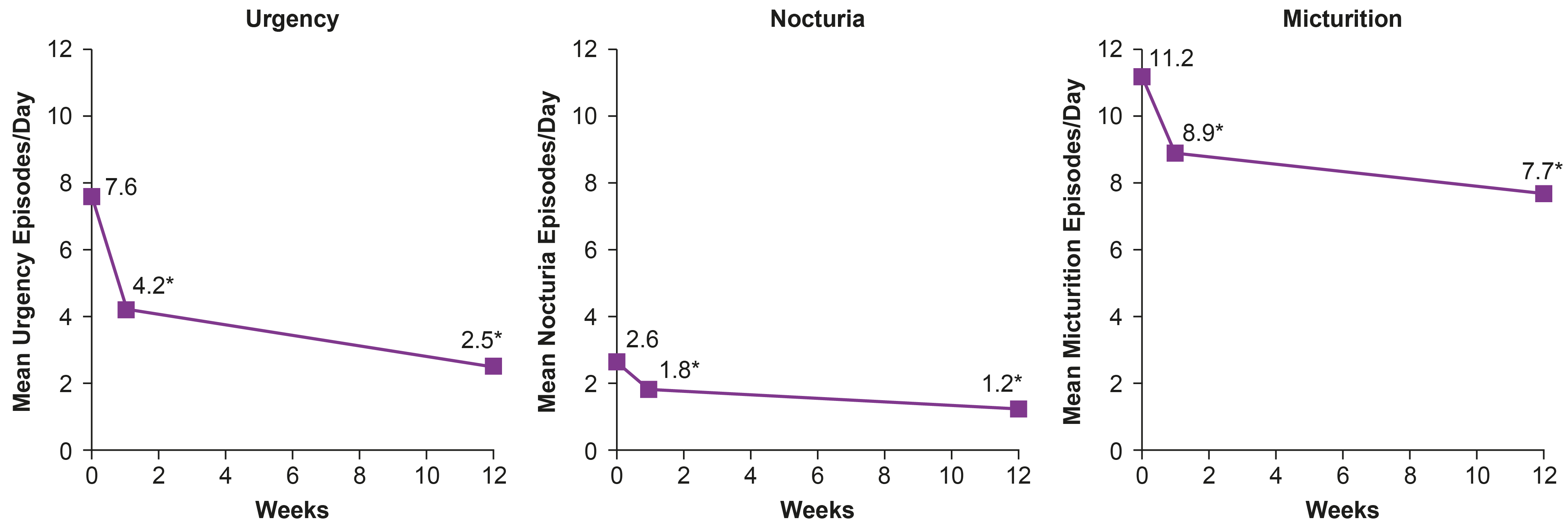

Supplement: Supplementary file 3 — High-resolution image (TIF 933 kb) [file 192_2020_4423_MOESM2_ESM.tif]
